# An Infant with Congenital Diaphragmatic Eventration with Dextrocardia: A Case Report

**DOI:** 10.31729/jnma.7029

**Published:** 2022-03-31

**Authors:** Ramana Rajkarnikar, Rabin Thami, Priyanka Dahal, Robal Lacaul, Rashmi Shrestha

**Affiliations:** 1 Department of Surgery, Kanti Children's Hospital, Maharajgunj, Kathmandu, Nepal; 2 Patan Academy of Health Sciences, Lagankhel, Lalitpur, Nepal

**Keywords:** *dextrocardia*, *diaphragm*, *diaphragmatic eventration*

## Abstract

Diaphragmatic eventration is a rare condition, and its association with dextrocardia is even a rarer clinical entity. Patients are usually asymptomatic, but the typical features include rapid breathing and recurrent respiratory infections. Here we present a rare case of a seven months old infant, who presented with cough, noisy breathing and chest retraction. The patient was diagnosed to have dextrocardia with diaphragmatic eventration with pneumonia by chest imaging and was treated in coordination with the medical team for underlying pneumonia. Afterwards, plication of the diaphragm was done through the trans-abdominal approach and the symptoms gradually improved postoperatively. For dextrocardia, since there were no structural abnormalities, the patient was kept in regular follow-up in the pediatric cardiology unit. Though most patients are asymptomatic, diaphragmatic eventration increases the risk of recurrent chest infection and hampers the quality of life of the patient, so timely diagnosis and intervention will greatly improve their quality of life.

## INTRODUCTION

Diaphragmatic eventration refers to an abnormal contour of the diaphragmatic dome with no disruption to the diaphragmatic continuity.^1^ It can be congenital or acquired. Congenital diaphragmatic eventration is rare (prevalence <0.05%) in which the diaphragmatic muscle is replaced by fibrous-elastic tissue leading to a thinned and pliable central portion of the diaphragm.^2-5^ Dextrocardia is a rare congenital disorder in which the heart resides on the right side of the thoracic cavity and is often associated with other development anomalies. The association of diaphragmatic eventration with dextrocardia is exceptional.^1^ Here we present such a case of an infant with dextrocardia with diaphragmatic eventration.

## CASE REPORT

A seven-months male baby, second of the twin delivery born via normal vaginal delivery at term, presented with a history of chest retraction noticed since the first month of life, but did not seek any medical advice until around six months of age when chest retraction gradually increased and patient developed fast breathing and cough. The patient was also not gaining weight as compared to his twin brother. He was taken to a nearby hospital and referred to our centre, Kanti Children's Hospital for further evaluation. On examination, the patient was thinly built. There was no cyanosis, clubbing, pallor, or oedema. The patient was dyspneic with a respiratory rate of 54/min and SpO_2_ 84-85% in room air with other vital signs within normal limits. On chest examination, there was bilateral equal chest rise with bilateral intercostal as well as subcostal retraction, with the apex beat palpable at right 4^th^ intercostal space in the midclavicular line. On auscultation of the chest, a conducted sound was heard mostly on the left middle zone, first and second heart sound heard with no obvious murmur. Other systemic examination findings were within the normal limit.

**Figure 1:**
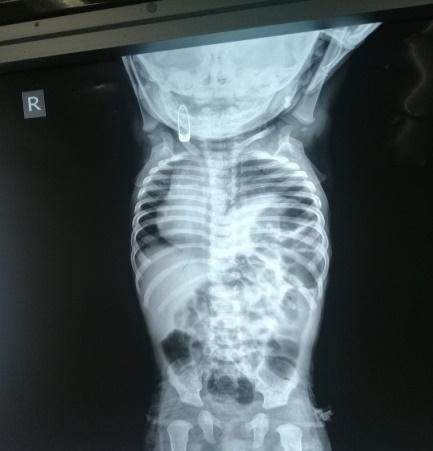
Chest X-ray showing elevated dome of left hemidiaphragm with cardiac silhouette towards the right side.

The patient was admitted in ward, and baseline investigations were sent, reports of which came within normal limits. His chest imaging revealed an elevated dome of the left hemidiaphragm with the apex of the cardiac silhouette towards the right side (Figure 1).

**Figure 2:**
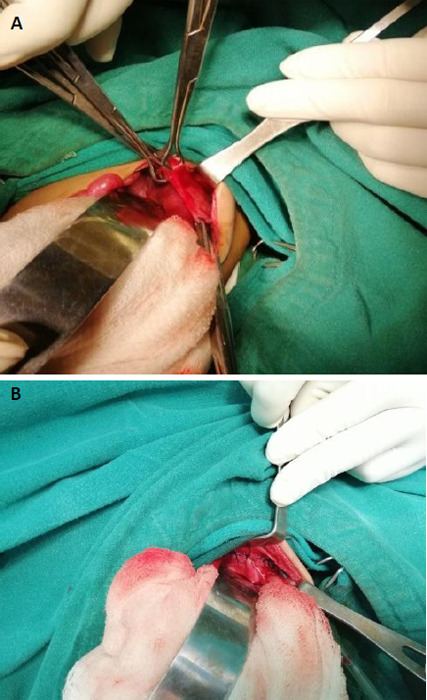
A) Intraoperative image of thinned out left hemidiaphragm, B) Image of the diaphragm after plication.

**Figure 3:**
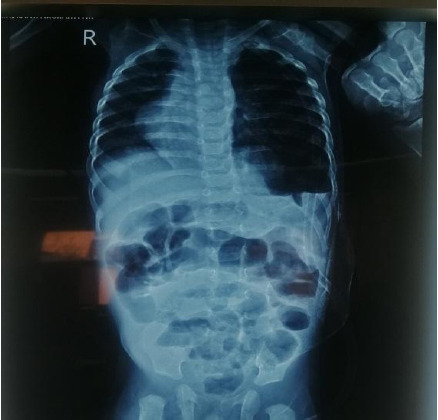
Post-operative chest X-ray showing lowered left hemidiaphragm with chest tube in-situ on the left side.

Echocardiography was done which revealed dextrocardia with no significant structural abnormalities. The patient was treated in coordination with the medical team for pneumonia. Once patient was maintaining oxygen saturation in room air, surgery was planned; plication of the diaphragm was done through the trans-abdominal approach (Figure 2). Intraoperatively the chest tube was kept in situ. The patient was kept in the Surgical Intensive Care Unit for two days. Post-operative chest x-ray showed a small pneumothorax on the left side with a chest tube in situ (Figure 3). Pneumothorax was resolved by the 2^nd^ postoperative day, then the chest tube was removed. The patient was then shifted to the surgical ward and oral feeding was started. Alternate-day dressing was done and parenteral antibiotics were continued for a total of seven days. Patient's chest retraction subsided and he was maintaining oxygen saturation in room air, he was then discharged on the 7^th^ postoperative day.

Paediatric cardiology consultation was done, and as there were no structural cardiac abnormalities, the patient was asked to be on follow-up on an outpatient basis.

## DISCUSSION

Eventration of the diaphragm is a condition in which all or part of the diaphragm is largely composed of fibrous tissue with only a few or no interspersed muscle fibres. Dextrocardia is a congenital condition in which the heart is located on the right side of the thorax. Diaphragmatic eventration is relatively rare and its association with dextrocardia has rarely been reported.^1-3^ It is also found to be associated with other anomalies such as hypoplastic lung, congenital heart disease and cryptorchidism.^2^

Eventration of the diaphragm is generally asymptomatic but symptoms related to the gastrointestinal tract, respiratory embarrassment, and rarely cardiac dysfunction, have been attributed.^1,4^ Typical symptoms included fast breathing and recurrent respiratory infections. In some patients with left diaphragmatic eventration, gastrointestinal symptoms like epigastric pain, vomiting and weight loss may be present.^5,6^

Dextrocardia, in most cases, is an incidental finding and is often associated with other development anomalies( cardiac and extracardiac).^6^ While assessing cases of diaphragmatic eventration, attention should be given to the nutritional status of the patient as well because undernourishment due to poor feeding may also be present.^2,4^

Diagnosis of diaphragmatic eventration can usually be made on standard posteroanterior, and lateral chest films, other modalities of imaging such as chest CT, fluoroscopy and ultrasonography are also used for diagnosis.^3, 7, 8^ However, in our case the diagnosis of dextrocardia with diaphragmatic eventration was established with chest x-ray and echocardiogram only.

In a patient with mild symptoms, non-surgical supportive care including chest physiotherapy and pulmonary rehabilitation is adequate. But if the symptoms persist, they should be managed surgically, plication is the treatment of choice.^2^ Surgical approaches can be thoracotomy, laparotomy and Video-Assisted Thoracoscopic Surgery.^4,8-10^ Compared with open surgery, thoracoscopic diaphragmatic plication has the advantages of a short operation time, less trauma, and rapid recovery as reported by Zhao S, et al.^11^

In our case, diaphragmatic plication was done through a trans-abdominal approach and the patient improved gradually without any complications. However, possible complications including pneumonia, dyspnea, pulmonary oedema, pleural effusion, and abdominal viscus injury may occur.^4,5,7^

Since dextrocardia with diaphragmatic eventration is a rare condition and it can be asymptomatic most of the time, clinicians need to have a high index of suspicion for its diagnosis. When an early diagnosis is made, surgical intervention such as diaphragmatic plication has shown to greatly improve symptoms and quality of life, especially in those who have recurrent respiratory infections. As in our case, the patient's symptoms gradually improved post-operatively and he was more comfortable, and that in turn was a relief for the parents.
